# Opportunities, Challenges, and Future Directions of Generative Artificial Intelligence in Medical Education: Scoping Review

**DOI:** 10.2196/48785

**Published:** 2023-10-20

**Authors:** Carl Preiksaitis, Christian Rose

**Affiliations:** 1 Department of Emergency Medicine Stanford University School of Medicine Palo Alto, CA United States

**Keywords:** medical education, artificial intelligence, ChatGPT, Bard, AI, educator, scoping, review, learner, generative

## Abstract

**Background:**

Generative artificial intelligence (AI) technologies are increasingly being utilized across various fields, with considerable interest and concern regarding their potential application in medical education. These technologies, such as Chat GPT and Bard, can generate new content and have a wide range of possible applications.

**Objective:**

This study aimed to synthesize the potential opportunities and limitations of generative AI in medical education. It sought to identify prevalent themes within recent literature regarding potential applications and challenges of generative AI in medical education and use these to guide future areas for exploration.

**Methods:**

We conducted a scoping review, following the framework by Arksey and O'Malley, of English language articles published from 2022 onward that discussed generative AI in the context of medical education. A literature search was performed using PubMed, Web of Science, and Google Scholar databases. We screened articles for inclusion, extracted data from relevant studies, and completed a quantitative and qualitative synthesis of the data.

**Results:**

Thematic analysis revealed diverse potential applications for generative AI in medical education, including self-directed learning, simulation scenarios, and writing assistance. However, the literature also highlighted significant challenges, such as issues with academic integrity, data accuracy, and potential detriments to learning. Based on these themes and the current state of the literature, we propose the following 3 key areas for investigation: developing learners’ skills to evaluate AI critically, rethinking assessment methodology, and studying human-AI interactions.

**Conclusions:**

The integration of generative AI in medical education presents exciting opportunities, alongside considerable challenges. There is a need to develop new skills and competencies related to AI as well as thoughtful, nuanced approaches to examine the growing use of generative AI in medical education.

## Introduction

As generative artificial intelligence (AI) technologies like Chat GPT and Bard gain prominence (Table 1), their potential applications and implications for medical education are attracting widespread attention [1]. Initially devised as experimental tools to test and hone AI technology, these systems are now being explored for practical applications with broad possibilities [2].

**Table 1:** Publicly available generative artificial intelligence (AI) services based on large language models.

Institution	Interface	Model	Notes
Open AI	Chat GPT	GPT-4	Most advanced publicly available model
BigScience	Hugging Face	BLOOM	Open-source model
Alphabet (Google)	Bard	LaMDA	Currently still labeled as “experimental”
Anthropic	Claude	AnthropicLM	Model trained on “constitutional” principles with the goal of enhanced safety
Stanford	Alpaca	LLaMA (Meta)	Much smaller than other models and able to run locally

Generative AI, a branch of machine learning capable of crafting new content in a variety of forms like text, images, audio, computer code, and video is finding applications in many fields [2]. Yet, harnessing this technology effectively, ethically, and equitably remains a challenge [3]. With the rapid integration of AI into various aspects of health care delivery, its infiltration into medical education seems imminent [4,5]. This intersection has sparked intense discussions and conjectures about the future of AI in medical education, revolving around its potential uses and limitations.

The integration of such a transformative technology into existing educational practices demands an informed, considerate approach. It necessitates not only an understanding of the capabilities and limitations of AI but also a forward-thinking blueprint for medical educators. This paper aimed to offer a comprehensive overview of the potential opportunities and challenges that generative AI presents for medical education. We conducted a scoping review of the available literature discussing generative AI in the context of medical education and distilled common themes of the proposed risks and benefits. Through this, we aimed to identify key areas for future exploration and deliberation, anticipating the continued growth of generative AI in medical education.

## Methods

### Overview

This study adhered to the standard scoping review framework proposed by Arksey and O’Malley [6]. We aimed to answer the primary research question: “What key themes emerge from the recent literature discussing the potential benefits and limitations of generative AI in medical education?” Our goal was to identify themes within recent literature related to potential applications and challenges associated with generative AI in medical education, with the hope of guiding future research. In the context of a state-of-the-art review, our focus was predominantly on literature published following the widespread adoption of generative transformer models such as ChatGPT. Accordingly, we limited our search to articles published from 2022 onward that specifically address generative AI, defined as AI capable of creating original content in multiple forms, including text, audio, images, and computer code. Our protocol is available in Multimedia Appendix 1.

### Identifying Relevant Studies

Our search strategy (Multimedia Appendix 2) encompassed both keywords and medical subject headings pertinent to generative AI and medical education combined using Boolean operators. We searched the PubMed, Web of Science, and Google Scholar databases for English language articles published from January 1, 2022, to June 21, 2023.

### Study Selection

Citations were managed using Covidence online software (Veritas Health Innovation). The first 100 articles were independently screened by both authors based on their titles and abstracts. This yielded substantial agreement (Cohen kappa=0.76). One author (CP) screened the remaining studies. The authors collectively refined the inclusion and exclusion criteria after initial title and abstract screening. CP then undertook full-text screening adhering to these criteria. A random subset of full-text articles was independently reviewed by CR. Conflicts at each stage were resolved through discussion and consensus.

Inclusion criteria required that articles discuss generative AI in the context of medical education. Articles were excluded if they exclusively focused on nonphysician education (such as nursing or dentistry), general AI topics in educational curricula, or nongenerative forms of AI (like predictive analytics and natural language processing).

### Charting the Data

Data abstraction was independently conducted using a structured form to capture article details, proposed uses for generative AI in medical education, potential limitations, and future recommendations. The authors convened to ensure consistency and resolve any disagreements.

### Collating, Summarizing, and Reporting the Results

Descriptive statistics were used to summarize study demographics. Qualitative data from the extraction forms underwent thematic analysis guided by the methodology by Braun and Clarke [7]. This involved open coding of the initial content from the extraction forms, the creation of axial codes that categorized existing codes, and subsequent recoding of data into identified themes and subthemes focusing on potential applications and limitations of generative AI in medical education (Table 2). To develop recommendations for research areas, we reviewed our themes as well as the existing literature and engaged in discussions with ourselves and other educators to contemplate areas for further exploration.

**Table 2:** Major themes identified, associated subthemes, and representative quotations.

Themes and subthemes		Representative quotations
**Theme 1: Test performance and preparation**		
	Licensing examination performance	“...we evaluated the performance of ChatGPT, a language-based AI [artificial intelligence], on the United States Medical Licensing Exam (USMLE). The USMLE is a set of three standardized tests of expert-level knowledge, which are required for medical licensure in the United States. We found that ChatGPT performed at or near the passing threshold of 60% accuracy.” [8]
	Specialty exam performance	“We challenged it to answer questions from a more demanding, post-graduate exam—the European Exam in Core Cardiology (EECC), the final exam for the completion of specialty training in Cardiology in many countries. Our results demonstrate that ChatGPT succeeds in the EECC.” [9]
	Undergraduate exam performance	“It can be concluded that ChatGPT helps in seeking answers for higher-order reasoning questions in medical biochemistry.” [10]
	Improving understanding	“Moreover, active surgeons who completed their training over a decade ago may find LLMs [large language models] helpful for continuous medical education (CME)...By utilizing an up-to-date LLM as a supplementary resource in their decision-making process, surgeons may have additional means to stay informed and strive for evidence-based care in their patient management.” [11]
	Self-directed learning	“Self-directed learning with ChatGPT can be phenomenal since it incorporates multiple domains and learns from the conversation it has with the student.” [12]
	Exam preparation/practice	“However, ChatGPT performed acceptably in negative phrase questions, mutually exclusive questions, and case scenario questions, and it can be a helpful tool for learning and exam preparation.” [13]
**Theme 2: Novel learning strategies**		
	Development of personalized learning plans	“The creation of personalized quizzes for students is an illustration of the use of generative AI in medical education evaluations. By analyzing each student's strengths and weaknesses, generative AI can generate unique formative and summative assessments for each student.” [14]
	Creation of learning materials	“Language models can analyze the performance of individual students and generate personalized learning materials that address their specific areas of weakness. For example, if a student struggles with a particular medical concept, the language model can generate additional resources or exercises to help them better understand it.” [1]
	Providing feedback	“By serving as a virtual teaching assistant, ChatGPT could be leveraged to provide students with real-time and personalized feedback.” [15]
	Communication skills training	“Although in its infancy, AI chatbot use has the potential to disrupt how we teach medical students and graduate medical residents communication skills in outpatient and hospital settings.” [16]
	Clinical image generation for learning	“...text-to-picture AI system is a developing and promising tool for medical education…With the use of ‘non existing people’ we can, with a good conscience, provide image material whose dissemination on the internet or social media does not violate patients’ privacy.” [17]
	Medical humanities exercises	“In a small-group educational setting, students will have the ability to create art that may tell a patient’s story, help in debriefing, and share an experience with others.” [18]
**Theme 3: Writing and research assistance**		
	Assisting non-native speakers	“In this context, LLMs could be used to translate and correct manuscripts in ways that could reduce language barriers, thereby allowing scholarly work from non-native English-speaking countries to be considered on a more equal footing.” [19]
	Translations	ChatGPT’s ability to translate language effectively can be utilized by medical professionals and educators to help communicate with patients from different linguistic backgrounds, in order to provide the best medical care.” [20]
	Literature review/summarization	“...medical researchers can use GLMs [generative language models] to scan and analyze vast amounts of medical literature quickly, identifying relevant studies and summarizing their findings. This can significantly reduce the time spent on literature reviews, allowing researchers to focus more on their primary research work.” [14]
	Fabricated references/hallucinations	“Simply put: ChatGPT generates fake citations and references.” [21]
**Theme 4: Academic integrity concerns**		
	Cheating on examinations	“The ability of LLMs to respond to short-answer and multiple-choice exam questions can be exploited for cheating purposes.” [22]
	Reduced effectiveness of learning exercises	“Student dependency on the language model may also propagate academic dishonesty or ‘cheating.’ For example, a student might use ChatGPT to complete an essay or other written assignment without fully understanding the material or putting in the required effort.” [15]
	Technological plagiarism	“Some educators are changing their course, examination, and grading structure and updating their definition of plagiarism to include, ‘using text written by a generation system as one’s own (eg, entering a prompt into an AI tool and using the output in a paper).’” [23]
	Need for policy development	“Consensus-based guidelines at the institutional and/or national level should be implemented to govern the appropriate use of [generative artificial intelligence].” [24]
	Guidance for disclosure and transparency	“Emerging issues have been raised with technology-generated academic papers, including how to define the extent of using AI assisted editing, the way of disclosure, privacy and confidentiality, and boundary of integrity.” [25]
**Theme 5: Accuracy and dependability**		
	Reliance on training data	“Although ChatGPT is trained on large amounts of data, there is always the possibility of errors or oversights in its training process, and the training data itself may contain inaccurate information.” [15]
	Lack of up-to-date information	“...the data set that ChatGPT was trained on was last updated in 2021. As a result, it is possible that the system is not able to provide users with the most up-to-date information, decreasing its reliability.” [26]
	Hallucination	“ChatGPT repeats its answers with much confidence and clear explanations, even in case of a totally wrong answer. This is technically called hallucination.” [27]
	Confidence expressed by models	“ChatGPT, with apparent confidence, provided an essay on liver involvement which, in reality, has not been reported yet.” [28]
	Misinformation propagation	“Further, AI-generated content can potentially produce misinformation or biased information...” [14]
	Limited accuracy in specific areas	“Consequently, the current level of accuracy is not yet sufficient for immediate clinical application in patient care.” [11]
	Need for further training in limitations	“AI is still underrepresented in the medical curriculum, and students lack the opportunity to engage more intensively with the topic of AI and develop the required expertise.” [29]
**Theme 6: Potential detriments to learning**		
	Overdependence	“Lastly, there is a need to delve deeper into the possible consequences of overdependence on LLMs in medical education.” [22]
	Challenges with assessment	“The performance of AI on certification tests says as much about the nature of those assessments as it does about the remarkable capacity of AI to pass them. We need to think carefully about the kind of performance we want our assessments to elicit.” [30]
	Propagating inaccurate information	“...students may find it challenging to differentiate between genuine knowledge and unverified information. As a result, they may not scrutinize the validity of information and end up believing inaccurate or deceptive information.” [22]
	Inequities in access	“Generative AI tools and LLMs may increase the inequity among students and educators, given that these tools are not equally accessible to all of them.” [22]

## Results

### Study Characteristics

**Figure 1:**
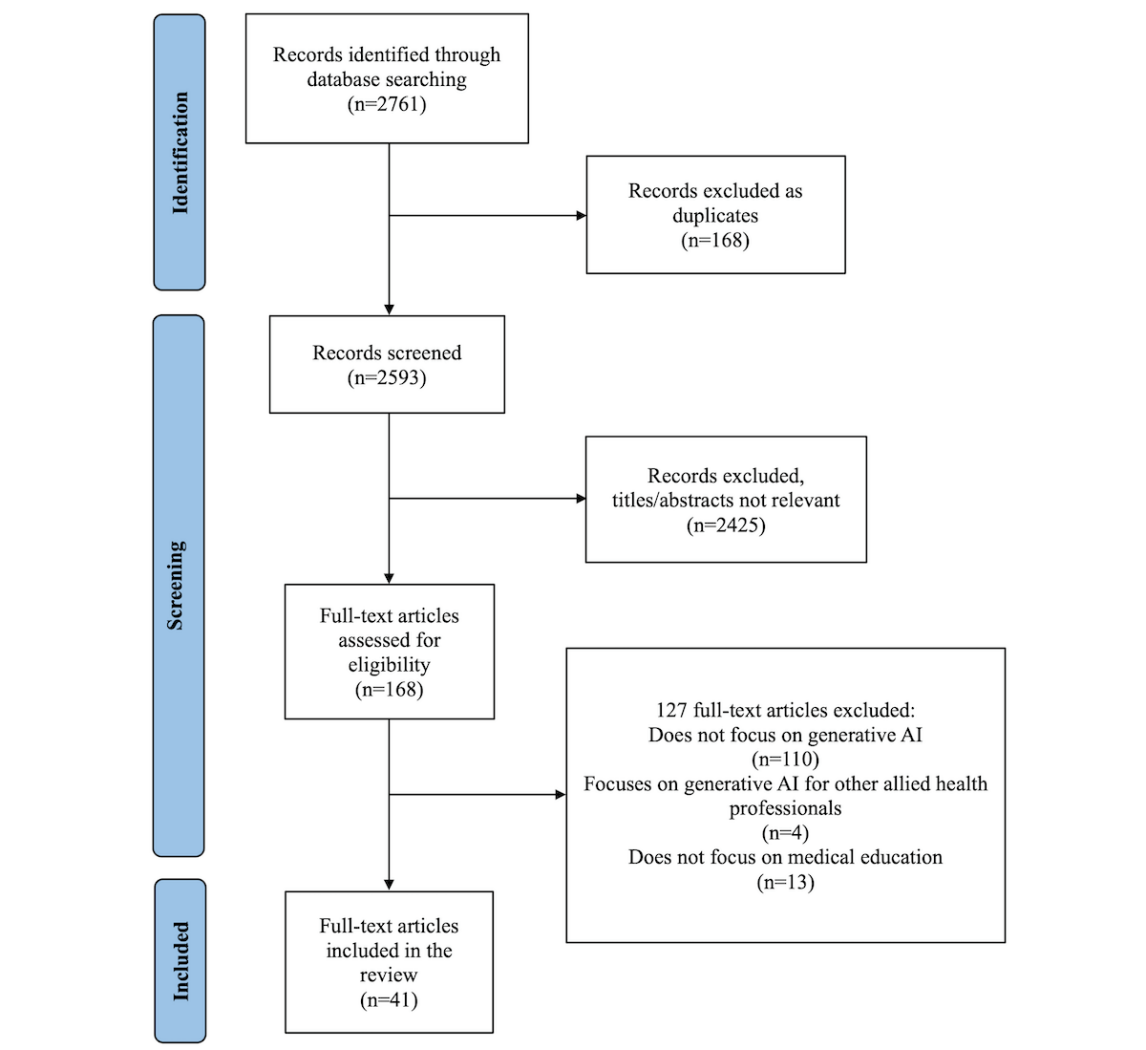
PRISMA (Preferred Reporting Items in Systemic Reviews and Meta-Analyses) flow diagram of search and screening for generative artificial intelligence (AI) in medical education articles.

Our initial search identified 2761 unique titles (Figure 1). After removing 168 duplicates, 2593 studies were available for screening. Of these, we found 2425 to be unrelated to our specific research focus, and we excluded another 127 studies for not focusing specifically on generative AI in medical education or for discussing a nonphysician population. A total of 41 articles were included in our final analysis.

In terms of article type, a slight majority were opinion pieces (21/41, 51.2%), with the remaining being original research articles (20/41, 48.8%). Of these original research articles, 16 reported on the performance of generative AI in standardized assessments within the field of medical education. Notably, all the studies included in our analysis were published within the year 2023.

### Potential Benefits of Generative AI in Medical Education

#### Test Performance and Preparation

Several studies focused on the role of generative AI models in tests of medical knowledge [8-11,13,26,27,31-39]. These examinations ranged from general medical knowledge tests such as the United States Medical Licensing Exam to specialized examinations in fields like cardiology, neurology, and ophthalmology [8,9,33,37,38]. Additionally, the performance of this technology has been analyzed in undergraduate subjects such as parasitology and biochemistry [10,32].

Overall, generative AI models showed impressive performance on standardized tests, though there were instances where they failed to pass certain exams, such as Taiwan's Family Medicine Board Exam [13]. Only a handful of these studies delved into the potential implications of generative AI's performance on these tests [8,33]. Those that did posited that this technology could be useful for self-directed learning or exam preparation [8,11,34]. However, none of these studies provided an explicit exploration of this process.

#### Novel Learning Strategies Through Generative AI

Numerous studies underscored the potential of these AI models to adapt to individual learners' requirements, offering a customized learning experience [1,14,15,20,22,34]. The development of personalized learning plans and learning materials as well as providing tailored feedback to learners are suggested potential avenues for exploration [1,14,15,20,22,34].

Several studies showcased initial examples of innovative teaching methods using generative AI. For instance, Webb [16] discussed the potential for generative AI to enhance communication skills for emergency medicine physicians, particularly for delivering difficult news. This was achieved by simulating patient reactions and dialogues during the disclosure of a new cancer diagnosis [16].

AI image generation technology has also been used in 2 distinct studies [17,18]. The first application involved generating images for case-based learning in plastic surgery, for which AI-produced photographs of conditions like skin tumors were used [17]. The second study suggested using AI-generated images for reflective exercises within a medical humanities curriculum [18].

Both papers emphasized that the use of AI-generated images could alleviate concerns surrounding copyright infringement or patient privacy that are inherent in using clinical photos or human-created artwork. Additionally, other papers provided instances of AI-generated content to demonstrate the potential for creating novel learning materials with this technology. However, the range of examples provided in the current literature is relatively limited [1,12,15,31].

#### Writing and Research Assistance Through Generative AI

Several authors discussed the use of generative AI as a potential writing or research aid [19,22,23,25,28,40]. They suggest that this technology could assist non-native English speakers with improving their writing proficiency as well as provide more comprehensive translation of foreign language content.

Numerous articles underscored the potential of generative AI to assist with literature reviews and summarizations [1,12,14,20,22,25]. However, they cautioned against the possibility of generative AI fabricating references and information, a pitfall commonly referred to as “hallucination.” This issue was brought to the fore in a piece by the editor of Medical Teacher, which recounted the journal’s first encounter with a “hallucinated” citation in a manuscript submitted for publication [21].

This article, along with others, highlights the potential for unethical practices, such as presenting AI-generated work as human-authored, and underscores the need for awareness and integrity when using these tools [12,14,15,19,20,22,23,25,40-43].

### Potential Limitations of Generative AI in Medical Education

#### Academic Integrity Concerns

As touched upon in the preceding paragraph, a significant worry cited by numerous authors is the potential threat to academic integrity and the possible misuse of this technology [12,14,15,19,20,22,23,25,40-43]. Many of the prospective advantages of generative AI can also be seen as potential pathways for unethical practices. For instance, generative AI could be used to dishonestly improve performance on examinations or assessments, misrepresent AI-generated text as written by a human, or circumvent traditional learning exercises designed for skill development [12,14,15,19,20,22-25,40-43].

Many authors emphasize the need for establishing clear-cut policies on the acceptable uses of generative AI within the realm of medical education [14,22,40,42,43]. These should outline the circumstances under which this technology can be utilized and also provide guidance on its disclosure in scholarly publications [21,40,43]. The creation of such policies would aim to maintain integrity and promote responsible use of this technology in the educational context.

#### Accuracy and Dependability

The precision and trustworthiness of generative AI are fundamental concerns thoroughly elaborated in many publications [8,11-15,20,22,24,32,35,41,42]. Several authors underscore that the knowledge base of these models is constrained by their training data, given that most models lack internet access to retrieve the most current information [10,22,34,37,44]. The tendency of these systems to produce nonexistent references presents a substantial issue, and it can be challenging to discern when an AI system is generating misleading or inaccurate data [1,21,25,27,28]. This is due to the unwarranted confidence often accompanying these fallacious outputs, which does not truly reflect the accuracy of results [45].

The propensity of these systems to generate and propagate misinformation is a notable risk. Despite the remarkable performance of these models on standardized tests, they still commit significant errors, and their performance is often on par with that of novice learners [32,35,36]. Various studies raise concerns regarding model bias and the potential for perpetuating stereotypes [14,15,19,22]. The majority of the authors stress the need for heightened awareness among educators and students regarding these potential limitations. They further encourage vigilant and critical use of AI-generated data, promoting an attitude of informed skepticism.

#### Potential Detriments to Learning From Generative AI

Several publications highlighted the risk of generative AI adversely impacting the learning process. An overdependence on this technology could potentially curtail learners’ capacities for critical thinking and intricate problem-solving [15,24,25,36]. As AI usage becomes increasingly prevalent among learners, there may be a need to adapt assessment methods, given the potential effects on the validity of knowledge evaluations [30,46].

Furthermore, an overemphasis on AI-based learning opportunities could diminish human interaction and engagement, which are fundamental to learning and honing patient-interaction skills [22,47]. The allure of using generative AI as a principal source of knowledge may inadvertently disseminate incorrect medical information. Thus, a balanced approach to incorporating AI in the learning process becomes essential to safeguard against such potential pitfalls.

## Discussion

### Overview

This review offers a comprehensive summary of the latest research exploring the potential advantages and limitations of generative AI in the field of medical education. The analysis is organized into major themes that have consistently emerged in the literature. Given that all the included studies were published in 2023, this reflects both the novelty of this technology and its burgeoning use in medical education.

Although we have presented the benefits and limitations separately, there is potential for interaction between these elements that may amplify or moderate their individual impacts. Certain benefits may be synergistic, such as using standardized test data to generate personalized learning plans that target knowledge gaps or leveraging AI’s writing capabilities to synthesize the latest medical research into timely educational content. Some benefits might also help mitigate other limitations. For instance, using AI as a writing aid could strengthen learners’ skills in organizing and expressing their own ideas, instead of copying and pasting from other sources, making them less prone to academic misconduct. Generating novel images or materials through AI provides opportunities to consciously create more diverse and unbiased content than curating existing human-made materials. Conversely, the limitations could augment some of the benefits. Greater awareness of the accuracy limitations of AI and potential for hallucination could encourage learners to develop more conceptual models of understanding content or to consult additional resources to verify accuracy, thereby inspiring further, deeper learning. Further research should explore the complex dynamics between the advantages and disadvantages of AI in medical education given that each offers promise and peril. A nuanced perspective examining how benefits and limitations intersect will allow the realization of AI’s educational potential while proactively addressing its risks.

The articles uncovered in our review further demonstrate the need for additional research. Most studies tend toward speculation or opinion pieces. There currently is an absence of empirical research examining the practical application and assessment of this technology with learners. To ensure this research yields actionable results, formulating appropriate research questions is paramount.

**Figure 2:**
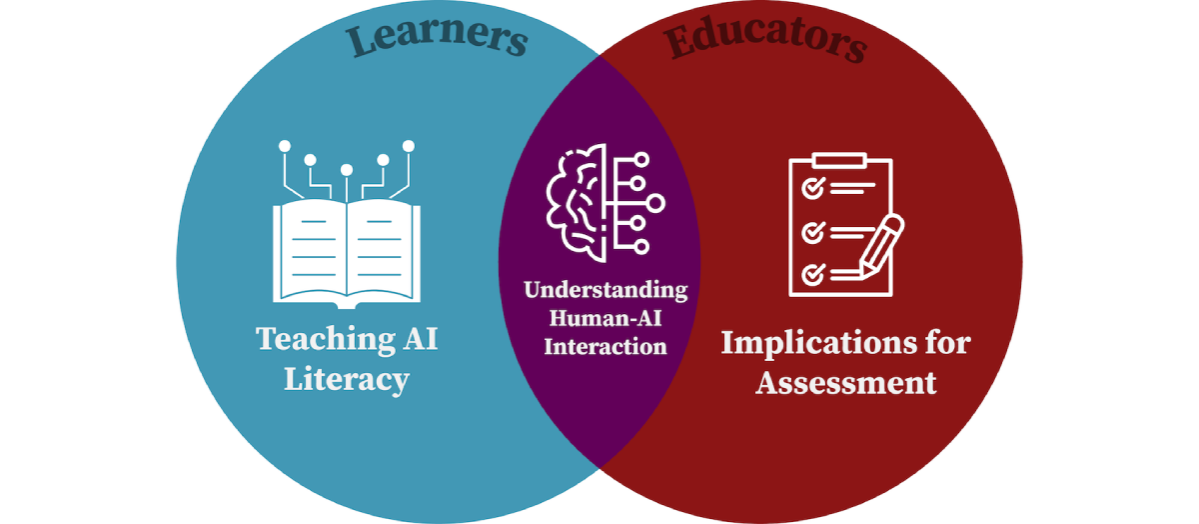
Proposed areas of investigation focused on learners, educators, and relevant to both. AI: artificial intelligence.

We propose the following 3 main areas of investigation relevant to learners, educators, and both: (1) improving learners’ AI literacy, (2) considering implications for assessment, and (3) exploring human-AI interaction (Figure 2).

### Area of Investigation for Learners: A New Literacy

In our estimation, the largest issue related to learners with AI is developing what has been called AI literacy. Within health profession education, AI literacy encompasses understanding the capabilities of AI; integrating AI into practice; and ensuring inclusion, equity, and responsible use of AI [48]. Several papers underscore the importance of developing new skills and competencies related to AI [14,19,42,43]. Although AI-related education is gaining momentum in medical schools, we found no curricula specifically focusing on generative AI. Similarly, we identified only 1 study examining learner attitudes toward generative AI in medical education [29]. The authors noted generally positive opinions albeit limited by unfamiliarity with these tools. A key component in developing curricula for learners related to AI will be a comprehensive needs assessment, including an assessment of attitudes. As one paper remarked, “it cannot be assumed that the generation of people who have grown up with digital technologies and are proficient in their use are also aware of all the options and ethical consequences of the use of new technology in their professional field” [29]. We would extend this perspective to include that we cannot assume knowledge of the technical limitations of new technology either.

Therefore, it makes sense that many of the skills highlighted as important for learners stem from potential constraints or concerns associated with this technology. A significant issue lies in data accuracy, with many authors drawing attention to this technology’s propensity to “hallucinate,” or create false information, and its knowledge being confined to the training data set [1,10,21,22,25,27,28,34,37,44]. Moreover, concerns have arisen that generative AI may produce biased content or lack representation of all populations [8,11-15,19,20,22,24,32,35,41,42]. These concerns point toward the need for curricula that equip learners with the knowledge to use this technology effectively, ethically, and responsibly. However, making users aware of these concerns is merely the first step toward addressing them. Determining the accuracy and quality of any source is a crucial skill, and medical education should foster critical appraisal skills for both primary and secondary medical literature (digital or otherwise), typically involving author credibility assessment, source evaluation, and external vetting. Generative AI, however, poses a challenge as it is difficult to assess in terms of credibility, can convincingly create sources, and seldom generates identical answers to questions.

This inability to observe how a response is generated is often referred to as “the black box” problem [49]. If traditional methods cannot be used to verify the accuracy of generative AI responses, we might initially think we need a new approach to train learners to effectively interact with this technology. However, we should consider how skills we already emphasize can be applied in this new context. Black boxes are not exclusive to AI, and ambiguity is frequently encountered in clinical settings. Dealing with medical enigmas such as unusual disease presentations; unexplained lab results; and information quality from a consulting physician, textbook, or manuscript are all “black boxes” to which we must grow accustomed in medicine. Therefore, although how to use AI safely and effectively is a new problem, the underlying skills are familiar to medical educators. Becoming comfortable navigating the uncertainties of AI technology likely will aid learners as they encounter similar challenges in the clinical environment.

Data uncertainty can be viewed from a positivist perspective with error margins and reliability estimates or from a pragmatic perspective, which focuses on the data’s utility [50]. Instead of focusing on teaching learners to verify the accuracy of AI-generated information, we should prompt them to consider the more crucial question of what actions these data may inspire. Learning about AI interactions may shed light on how we engage with other artifacts or individuals in the clinical environment, compelling learners to ponder what “accuracy” means in a clinical or learning context [51]. As part of a curriculum, it might be beneficial to have learners gain expertise in navigating hard-to-verify information and train them to construct valid arguments for their conclusions. The tensions of navigating information provided by technology and other sources are fertile ground for exploration and discussion among learners, particularly as AI begins to drive more clinical decisions [4].

Similarly, missing or incomplete data in generative AI models are often cited as a limitation; however, it is essential to consider the standard against which this is compared. To our knowledge, there is no comprehensive medical knowledge resource nor an agreed-upon metric for evaluating a resource’s comprehensiveness. Medical textbooks, often considered the gold standard in medical knowledge, are perpetually outdated, are limited in scope, and may contain inaccuracies [52-54]. Considering the primary medical literature, most published research findings are suggested to be false [55]. Thus, inaccurate or incomplete data are not a new issue but a problem we might only just be recognizing. Teaching learners to derive correct conclusions despite misleading, missing, or inaccurate data should be our primary focus.

These critical evaluation skills are also essential to dealing with issues surrounding bias and underrepresentation. Biases in generative AI are often suggested to be the result of training data, though this conclusion may be challenging to validate [56]. Much like data accuracy, data bias is not a new problem. Lack of representation and bias in medical records data are major concerns, and we are only beginning to recognize biases in technology that has been in use in health care for years [57-59]. Although we concur with recommendations to work toward minimizing and eradicating bias, complete elimination may not be feasible. Our focus should instead be on teaching ways to understand the effects of these biases and how to make patient care decisions when data or evidence may be biased. We again advocate for a pragmatic approach, equipping learners with strategies to understand how biased data can retain value while emphasizing the importance of recognizing both intended and unintended consequences.

In sum, we recommend further development and exploration of curricula designed to enhance learners’ AI literacy. However, the key areas of focus should be directed toward critical appraisal skills and navigating uncertainty. Focusing on these skills will have the benefit of applicability in the clinical environment and developing a foundational approach that will continue to be useful as technology rapidly changes.

### Area of Investigation for Educators: Implications for Assessment

Generative AI models’ impressive performances on diverse standardized assessments in medical education not only demonstrate the abilities of these tools but also suggest a reevaluation of our current assessment methods. This sentiment aligns with the viewpoint of Pearce and Chiavoroli [30] that we must rethink our learner assessment methods in a world where generative AI is increasingly prevalent. Even though the quality of these assessments might remain the same, their relevance needs reconsideration in an era when a chatbot can effortlessly provide answers to multiple choice questions.

Primarily, the objective of these assessments should be revisited. Formative assessments could potentially be reconceptualized as AI-enhanced learning opportunities. Here, the technology could offer explanations for the provided answers, or the learners might pose follow-up queries. For curriculum evaluation–based assessments, educators often aim to test learners’ capabilities to comprehend and perform higher-order cognitive skills [60,61]. In this context, AI’s capacity to mimic higher-order cognition in its responses can offer an insightful reference point for educators to reconsider their approaches to assessing understanding, application, and analysis, for example, and reassess their existing strategies [62]. Observing how generative AI responds to these queries could assist us to frame more incisive questions or even inspire us to refine our comprehension of human cognition.

Conversely, multiple authors underscore the possibility of bias and inaccuracies in AI systems [8,11-15,19,20,22,24,32,35,41,42]. Any assessment form that uses or is developed using AI must undergo rigorous pilot-testing, with comprehensive validity evidence collected, including an exploration of the implications of using this technology. AI is already being utilized in various significant decisions, such as medical school selection [63]. Although the focus tends to lean on the AI models’ task completion capabilities (or their performance in exams, as mentioned earlier), medical educators should also pay careful attention to how these uses affect humans.

Although we primarily discuss issues in assessment, we encourage educators to consider and examine how generative AI impacts our understanding of existing practices within medical education. Similarly, we should attune to and study the anticipated and unanticipated ways this technology will shape our field going forward.

### Area Common to Both: Understanding Human-AI Interaction

To adequately evaluate the impact of AI on educators and learners, we need to develop strategies that unravel the complexities intrinsic to human-AI interactions. A few studies outline potential scenarios in which educators or learners might interact with AI systems, such as in self-directed learning, simulation environments, and writing assistance [8,11,12,14,19,22,23,25,28,34,35,38,40,64]. These interactions permeate beyond the academic realm; for instance, a study by Gabrielson et al [44] addressed the utilization of AI for tasks like clinical care, patient communication, and administrative duties. Although literature tends to emphasize the technical aspects of these applications, the user’s role is critical in determining the potential success and limitations of these opportunities.

Although individual voices expressing enthusiasm or concern for this technology exist in the literature, the general attitudes of medical educators toward AI are not yet fully understood. A broader assessment of attitudes among both educators and learners toward generative AI is necessary. Although the results will likely hinge heavily on their familiarity with this technology, even minimal experience allows insight into how the diffusion of this technology will occur in practice to meet learners where they are. Ideally, novel AI applications in education should be accompanied by investigations into learners’ perceptions of this technology, as the success of AI-based educational interventions could largely depend on users’ attitudes toward and experiences with the AI system or AI technology in general. Any study reporting an AI-based educational innovation should include a comprehensive description and evaluation of contextual factors that might influence its success. Curriculum evaluation methodologies focusing on context, such as the Context, Input, Process, Product (CIPP) model, theory-driven evaluation, or realist evaluation, might be particularly adept at accounting for and examining human-AI interaction within an educational intervention [65].

Analogous to considering human-AI interaction in AI applications, we must also contemplate the influence of generative AI on learners and educators. Several articles voice concerns about potential academic dishonesty [12,14,15,19,20,22,23,25,40-43]. Instances of technological plagiarism already exist, in which AI has generated abstracts or entire scientific papers with minimal human involvement [66,67]. We should consider the impact of this new technology on the ethical values and professionalism of both learners and educators. Dependence on AI could potentially compromise learning opportunities or skill development that arises from task completion without assistance [15,24,25,36]. However, AI usage could redefine our understanding of what constitutes valuable skills for a physician. Many suggest that familiarity with AI technology should be incorporated into medical education, and we should investigate how teaching about AI usage affects our learners and educators [10,22,25,36].

Last, AI might influence human-human interaction. Multiple papers spotlight the development of writing skills, communication skills, and language translation as potential areas where AI could prove beneficial. An emerging field of AI-mediated communication focuses on AI’s influence on our interactions with others [68]. Existing tools like autocorrect and predictive text already impact our communication [69]. Several articles in our review underscore concerns with data privacy and trust. These amplified concerns, along with new AI-mediated capabilities to impersonate individuals or generate false content, might shape how we interact with others. If AI enhances our writing, the dynamics of our conversations could alter. However, not all outcomes are negative, as AI might facilitate broader dissemination or more seamless communication across language barriers [14,20,25].

### Limitations

This scoping review has several limitations that should be considered when interpreting the results. First, the search was restricted to articles published in English, which may have excluded some relevant non-English literature. The search was also limited to articles published from 2022 onward, given the focus on recent generative AI models. However, this excluded earlier literature on related topics like natural language processing in medical education. The thematic analysis process also has inherent subjectivity. Although we attempted to enhance trustworthiness through reflection and discussion, the themes generated represent our interpretation of the available literature.

The literature on generative AI in medical education is rapidly evolving, and new evidence may have emerged since our search was conducted. However, this scoping review provides a comprehensive summary of the key themes based on the available literature at the time of the search. The lack of empirical studies limits the ability to draw definitive conclusions regarding the actual impacts of generative AI on medical education. Most of the discussed benefits and challenges remain speculative. Further research investigating the real-world effects of integrating generative AI into medical curricula and practice is required.

### Conclusions

Generative AI brings transformative potential to medical education, but integrating it thoughtfully remains imperative. Although current literature speculates theoretically on AI’s prospects, empirical research is critical to guide effective, ethical implementation. Key areas needing investigation include developing learners’ skills to evaluate AI critically, rethinking assessment methodology, and studying human-AI interactions. Though AI offers exciting opportunities, like personalized learning and writing assistance, limitations around accuracy, bias, and dependence must be addressed through rigorous testing and curricula promoting responsible usage. Ultimately, realizing the full potential of generative AI in medical education requires focus not just on capabilities but also on impacts—aiming to augment human strengths while developing new competencies for interacting with emerging technologies. A thoughtful, balanced approach can allow AI to enhance medical learning while inspiring the creation of new knowledge, skills, and ways of thinking.

## Appendix

Multimedia Appendix 1 Review protocol.

Multimedia Appendix 2 Full search strategy.

Multimedia Appendix 3 PRISMA-ScR Checklist.

## Abbreviations

AI: artificial intelligence
CIPP: Context, Input, Process, Product
