# Supplementary material for: Opportunities, Challenges, and Future Directions of Generative Artificial Intelligence in Medical Education: Scoping Review
Source: JMIR Med Educ. 2023 Oct 20;9:e48785. doi: 10.2196/48785 (PMC10625095; doi:10.2196/48785)
Supplement: Multimedia Appendix 1 [file mededu_v9i1e48785_app1.docx]

Generative Artificial Intelligence in Medical Education– A Scoping Review Protocol

**Background**

As generative artificial intelligence (AI) technologies like Chat GPT and Bard gain prominence, their potential applications and implications for medical education are attracting widespread attention [1]. Initially devised as experimental tools to test and hone AI technology, these systems are now being explored for practical applications with broad possibilities [2].

Generative AI, a branch of machine learning capable of crafting new content in a variety of forms like text, images, audio, computer code, and video, is finding applications in many fields [2]. Yet, harnessing this technology effectively, ethically, and equitably remains a challenge [3]. With the rapid integration of AI into various aspects of healthcare delivery, its infiltration into medical education seems imminent [4,5]. This intersection has sparked intense discussions and conjectures about the future of AI in medical education, revolving around its potential uses and limitations.

The integration of such a transformative technology into existing educational practices demands an informed, considerate approach. It not only necessitates an understanding of the capabilities and limitations of AI but also a forward-thinking blueprint for medical educators. This paper aims to offer a comprehensive overview of the potential opportunities and challenges that generative AI presents for medical education. We conducted a scoping review of the available literature discussing generative AI in the context of medical education and distilled common themes in the proposed risks and benefits. Through this, we aim to identify key areas for future exploration and deliberation, anticipating the continued growth of generative AI in medical education.

**Objectives**

The primary objective of this review is to answer the question: “What key themes emerge from recent literature discussing the potential benefits and limitations of generative AI in medical education?” We aim to:

1. Identify the potential applications of generative AI in medical education.

2. Assess the challenges and limitations associated with the integration of generative AI.

3. Provide a roadmap for future research on this topic.

**Methods**

This scoping review will adhere to Arksey and O’Malley's framework, designed to provide a comprehensive overview of broad and complex topics [6].

**Eligibility Criteria**

We will include articles that:

- Discuss generative AI in the context of medical education.

- Are written in English.

- Are published from January 1, 2022, to June 21, 2023.

Exclusions will be articles that focus solely on non-physician education, general AI topics unrelated to medical education, or non-generative forms of AI.

**Search Strategy**

The search strategy will utilize keywords and medical subject headings relevant to generative AI and medical education. Databases include PubMed, Web of Science, and Google Scholar. This will be refined in consultation with a medical librarian.

**Study Selection**

Citations will be managed with Covidence online software. Two authors will independently screen the first 100 articles based on titles and abstracts, aiming for substantial agreement. One author will screen the remaining articles, and any conflicts will be resolved through discussion.

**Data Extraction**

Data will be extracted independently using a structured form to capture details about each article, proposed applications, limitations, and future recommendations. All authors will convene to discuss and resolve any inconsistencies in data extraction.

**Analysis**

Quantitative data will be summarized using descriptive statistics. Qualitative data will undergo thematic analysis according to Braun and Clarke’s methodology [7].

**Anticipated Outcomes**

We expect to identify various applications of generative AI in medical education, including but not limited to self-directed learning, simulation scenarios, and writing assistance. Challenges are likely to encompass academic integrity, data accuracy, and possible hindrances to learning. The review will culminate in proposing areas for future research and recommendations for educational strategy.

**Dissemination and Ethics**

Findings will be disseminated through peer-reviewed journals and academic conferences. As this study is a review of existing literature, ethical approval is not required.

**Conclusion**

This protocol provides a comprehensive methodology for a scoping review aimed at understanding the landscape of generative AI in medical education. Through this review, we aspire to provide a framework that assists medical educators in the thoughtful integration of AI into their teaching practices.
