# Supplementary material for: Opportunities, Challenges, and Future Directions of Generative Artificial Intelligence in Medical Education: Scoping Review
Source: JMIR Med Educ. 2023 Oct 20;9:e48785. doi: 10.2196/48785 (PMC10625095; doi:10.2196/48785)
Supplement: Multimedia Appendix 2 [file mededu_v9i1e48785_app2.docx]

**Multimedia Appendix 2: Full Search Strategy**

PubMed

((("Artificial Intelligence"[Mesh] OR "Generative AI"[All Fields] OR "Generative Model"[All Fields]) OR ("Artificial Intelligence"[Mesh] OR "Generative AI"[All Fields] OR "Generative Model"[All Fields]) OR (("Artificial Intelligence"[Mesh] OR "Generative AI"[All Fields] OR "Generative Model"[All Fields]) OR (Generative Model[All Fields] OR Generative Models[All Fields] OR Generative Models,[All Fields]) OR Generative AI[All Fields])) AND ((("Education, Medical"[Mesh] OR "Medical Education"[All Fields] OR "Teaching"[Mesh] OR "Learning"[Mesh]) OR (e-learning[All Fields] OR online education[All Fields] OR distance learning[All Fields] OR "blended learning"[All Fields]))

Web of Science

(TS=("Artificial Intelligence" OR "Generative AI" OR "Generative Model") AND TS=("Education, Medical" OR "Medical Education" OR "Teaching" OR "Learning" OR e-learning OR "online education" OR "distance learning" OR "blended learning"))

Google Scholar

("Artificial Intelligence" OR "Generative AI" OR "Generative Model") AND ("Education, Medical" OR "Medical Education" OR "Teaching" OR "Learning" OR e-learning OR "online education" OR "distance learning" OR "blended learning")
